# An unsupervised learning approach to find ovarian cancer genes through integration of biological data

**DOI:** 10.1186/1471-2164-16-S9-S3

**Published:** 2015-08-17

**Authors:** Christopher Ma, Yixin Chen, Dawn Wilkins, Xiang Chen, Jinghui Zhang

**Affiliations:** 1 Department of Computer and Information Science, Weir Hall, University of Mississippi, University, MS 38677, USA; 2 St. Jude Children's Research Hospital, Department of Computational Biology, Memphis, 262 Danny Thomas Place, Memphis, TN 38105-3678, Memphis, USA

**Keywords:** Markov process, eigenvector, gene-gene interaction

## Abstract

Cancer is a disease characterized largely by the accumulation of out-of-control somatic mutations during the lifetime of a patient. Distinguishing driver mutations from passenger mutations has posed a challenge in modern cancer research. With the advanced development of microarray experiments and clinical studies, a large numbers of candidate cancer genes have been extracted and distinguishing informative genes out of them is essential. As a matter of fact, we proposed to find the informative genes for cancer by using mutation data from ovarian cancers in our framework. In our model we utilized the patient gene mutation profile, gene expression data and gene gene interactions network to construct a graphical representation of genes and patients. Markov processes for mutation and patients are triggered separately. After this process, cancer genes are prioritized automatically by examining their scores at their stationary distributions in the eigenvector. Extensive experiments demonstrate that the integration of heterogeneous sources of information is essential in finding important cancer genes.

## Introduction

Understanding cancer biology and the mechanism behind cancer progression has always been an important branch of cancer research. Thanks to the advancement of computational technology, a huge amount of biological data such as microarray gene expression is readily available. Researchers make use of gene expression profiles to predict clinical outcome of breast cancer and identify several cancer subtypes. This can help in elucidating the association between several molecular levels which enable us to identify the biological relationships and understand the molecular processes driving the cancer. This could potentially lead to improvement in cancer diagnosis and patient survival analysis prediction. Consequently, other types of high throughput biological data are produced which are of great interest and as a matter of fact, we aim to integrate different sources of data in a unified framework with an objective to locate important biomarkers responsible for cancer progression through a ranking method.

Our framework is encoded with various heterogeneous sources of data including 1) Protein protein interaction (PPI) Network: Cancer is not a disease of individual mutation but a group of genes interacting together in a molecular network. Hence incorporating PPI network and pathway interaction information in cancer studies is critical in discovering interactions among genes and deciphering the molecular pathway of cancer. 2) Gene Expression profiles: DNA microarray-based technology has provided researchers ample opportunity to perform comprehensive molecular and genetic profiling of cancer by simultaneously studying how thousands of genes were being expressed in hundreds of patients. We used the gene expression data and performed the Pearson correlation coefficients calculation to determine the correlation of the gene expressions of various genes in a bid to identify co-expressed genes which are responsible for cancer development. 3) Patient Somatic Mutation Profiles: A table which records the mutation profile of each patient is also included in our framework as background information.

Separate Markov Chains on the genes and patients are defined and random walks are performed in order to obtain the results. Random walk based ranking work on cancer modules can be found in [2,4,5]. In [1], the authors utilize both the random walk and random walk with restart to rank genes with respect to their likelihood of being a member of each cancer module through the functional interaction network globally and interactions between genes in each cancer module locally. In [3], Erten, Bebek and Koyuturk investigate the topological similarity in PPI networks and suggest a random walk based algorithm to find genes with similar disease. They came up with a measure to calculate the topological profiles between the candidate genes and the driver genes. In [8], the authors utilize random walk and network community analysis for the identification of cancer-associated modules in gene expression data. In [9], Sharan, Ulitsky and Shamir survey a number of random walk related approaches, including direct methods and module-assisted methods. The algorithms propagate functional information and functional modules within the network, which are inferred for annotation purpose. Other techniques related to random walk are Markov field based propagation and Guassian Random Field propagation. The authors in [6,7] utilized those network propagation techniques in protein function prediction.

In [10], Zhang and Wei extend the general network propagation algorithm to consider graphs with nodes and edges to be positive and negative numbers for the sake of detecting differential gene expressions and DNA copy number variations (CNV). Gene up/down regulation or amplification/deletion CNV events are modelled to be positive and negative respectively. By exploiting the weighted connections between genes, gene labels are propagated sucessfully within the network. This method is capable of identifying hidden clusters to eliminate false positives and recover false negatives. However, it may also explore very weak similarities between genes as well. In our proposed random walk based framework, inspired by the algorithm of Google PageRank, randomness is introduced by permitting each gene to choose one patient to hop and each individual patient to choose one gene to hop randomly for minuscule amount of probability. In this method, both the gene nodes which are strongly connected and gene nodes with poor connectivity can also be exploited so as to discover biomakers globally through some noise introduced by random teleportation.

Our overall framework comprises five models which essentially differ from each other by the sequence and order the random walk is performed on our overall patient gene network. A multigraph is introduced in which the gene gene interaction network and the gene correlation network are merged thus multiple edges between a pair of gene nodes are allowed. Moreover, different sequences of traversal of this multigraph results in different transition matrices for the genes and patients respectively which culminates in our five different models. Our works sucessfully incorporates multiple heterogeneous data sources in a graph to find the cancer genes by computing the major eigenvector of each individual stationary matrix in each model and each gene is ranked in accordance with the corresponding value in the eigenvector rank. Comprehensive experiments demonstrate that the integration of heterogeneous sources of information is useful in discovering cancer genes and all six of the proposed models are able to rank those confirmed cancer genes as reported from other literature within top positions in the rank. The remainder of the paper is organized as follow: We will present the Methodology next. Afterwards, extensive experiments are performed and results are reported and tabulated. Lastly, we present conclusion and future work.

## Methods

In this section, we illustrate how to represent the three sources of information.

### Hetergeneous Sources of Data

1) Gene Gene Interaction: The gene gene interaction networks are encoded as an undirected graph *G*(*V, E*) where *V *stands for the genes and edges (*i, j*) *∈ E *are weighted by a weight matrix W, whose element *w_ij _*is the weight of the edge (*i, j*) *∈ E *which represents the strength of interaction between gene *i *and gene *j *using two sources of gene gene interaction networks described below. Two sources of protein protein interaction networks are utilized: PathwayCommons and HumanNet v.1. PathwayCommons is a database of biological pathway information compiled from multiple sources related to PPI interactions and functional relationships between genes in signalling pathways. Only human genes and interactions in Pathway-Commons are utilized in our framework. HumanNet is a probabilistic functional gene network constructed using naive Bayesian method to weigh different types of data evidence collected in humans, yeast and worms in accordance with their functionality in Homo-sapiens. A single interaction score is calculated as a result. HumanNet v.1 consists of 18,714 validated protein encoding genes in total.

2) Gene Expression Profiles: Gene expression is measured through high throughput microarray experiments which show the expression level of a gene on each person. The gene expression data show the expression levels of genes in both the tumor and normal samples which are used for evaluating the similarity between genes, where genes with similar gene expression are often perceived to also carry similar functionality. We capitalize on the gene expression data and construct a gene correlation graph/network. To summarize, a gene correlation graph/network is a graph H(V, E), where V represents genes and an edge (*i, j*) *∈ E *is weighted by calculating the Pearson correlation coefficient between their gene expression values.

3) Patient Mutation Profile: Patient-Mutation Profile is a two dimensional binary matrix with columns representing the genes and rows representing patients. Each entry is either 0 or 1, a 1 indicates that a mutation has occured in the tumor relative to the germline on that patient, a 0 otherwise.

#### Mutual Informative Model

**Figure 1:**
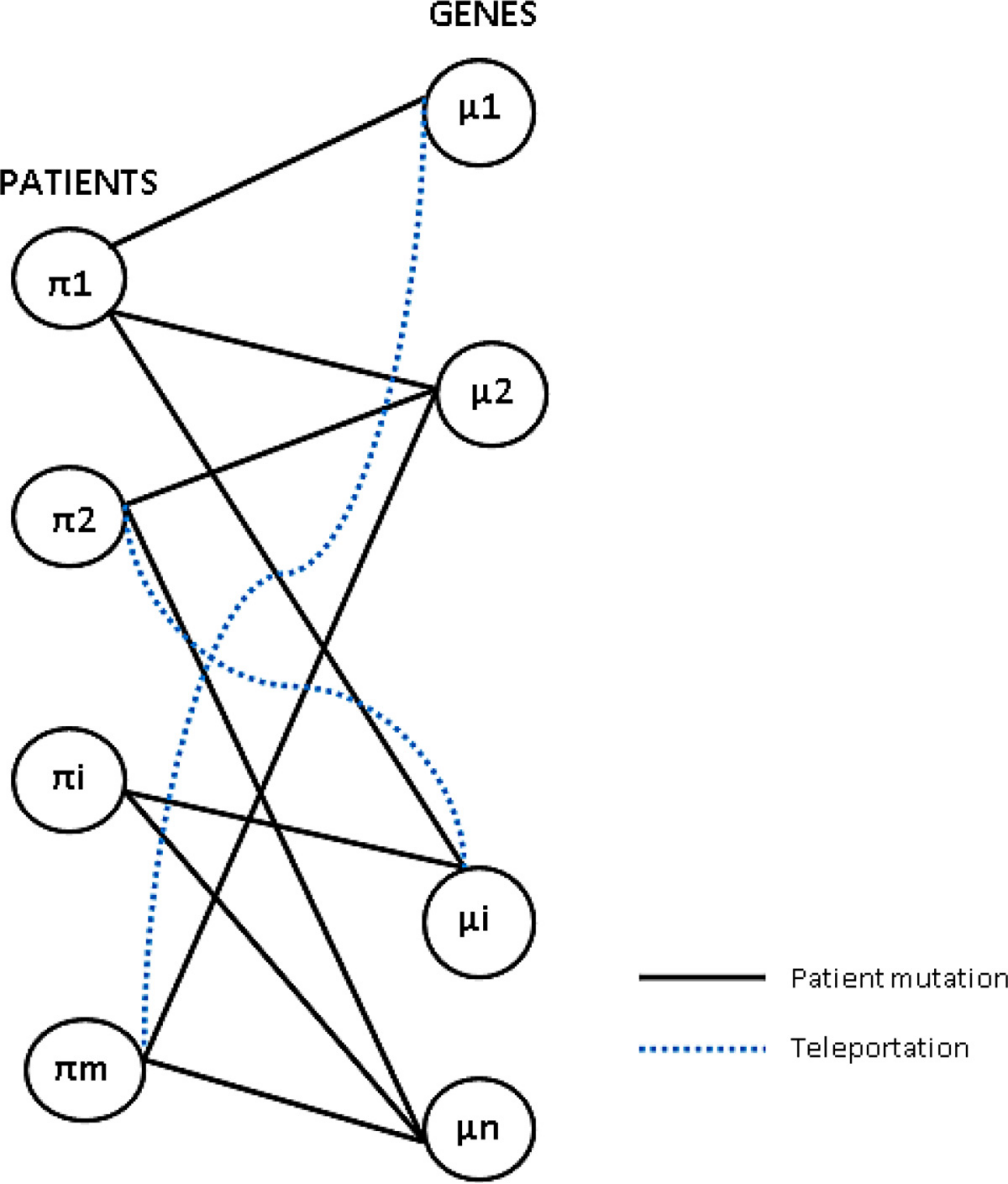
**Patient mutation network**.

In Mutual Reinforcement Model, (shown in Figure 1) each mutation (gene) is assigned a driver score *µ_i _*and each patient is assigned a patient score *π_i_*. Each patient is allowed to cast a vote on each mutation (gene) and vice versa. As a result, the driver score of a mutation (gene) is in proportion to the total votes the mutation (gene) received and the total votes received by the patient determine the patient score. Therefore, a high driver score means that the mutation is possessed by patients with high patient scores and a high patient score means that the patient possesses mutations with high driver scores. With the introduction of patient mutation profile, some notations can be laid out. Adjacency matrix *B *of a bipartite graph is defined as *B_ij _*= 1 if and only if patient *i *has gene *j *mutated and 0 otherwise we use *m *to represent the total numbers of patients and *n *the total genes numbers. As the driver score of a mutation (gene) *µ_i _*is directly prorportional to the number of patients possessing that mutation and the patient score *π_i _*is directly proportional to the total number of mutations possesed by the patient, the mutation score and patient score are mutually defined relative to each other and the equations below are justified.

$$\mu_{i}\infty \sum_{i\in l:B_{kj=1}}\pi_{i}$$

$$\pi_{i}\infty \sum_{j\in k:B_{ik}=1}\mu_{j}$$

To start with, the probability in which a mutation (gene) *j *traverses to patient *i *is defined by the following matrix:

$$B_{c}|i,j|=\frac{B_{ij}}{\overset{m}{\underset{k=1}{\sum }}B_{kj}}$$

Likewise, the probability in which a patient *i *traverses to mutation (gene) *j *is governed by the following matrix:

$$B_{r}|i,j|=\frac{B_{ij}}{\overset{n}{\underset{k=1}{\sum }}B_{ik}}$$

Notice that *B_r _*is a row stochastic matrix whereas *B_c _*is a column stochastic matrix. Randomness is introduced by allowing each patient to select arbitrarily a mutation (gene) to teleport for a small amount of time besides following the incident edges and hop to one of his neighbors in the gene partite set with the probability governed by matrix *B_r _*for most of the time. The factor 1 *− α *defines the probability in which the patient relinquishes the matrix *B_r _*for traversal and use teleportation for traversal. Since each mutation (gene) has equal probability to be chosen by each patient. The following transition matrix for patients is justified:

$$C_{r}|i,j|=\alpha *B_{r}+\left(1-\alpha \right)\frac{1}{n}I_{m*n}$$

where *I*_*m*∗*n *_is an *m *by *n *matrix with all entries equal 1. Likewise for mutations (genes), each mutation (gene) is allowed to select arbitraily a different patient to teleport for a small percentage of time and the following transition matrix for the mutations (genes) is defined.

$$C_{c}\left[i,j\right]=\alpha *B_{c}+\left(1-\alpha \right)\frac{1}{m}I_{m*n}$$

Next, we incorporate the gene gene interaction network and gene correlation network in our model for the mutation of genes. As described above, a gene gene interaction network can be encoded as an undirected graph *G*(*V, E*) where V represents the genes and edges (*i, j*) ∈ *E *are weighted by a weight matrix *W*, whose element *w_ij _*represents the interaction strength between gene *i *and gene *j*. Afterwards, normalization of matrix *W *is performed in order to define the transition probability matrix within the genes and hence the transition probability matrix *Q *= *D*^*−*1*W *^is a result. Note that *D *ia a diagonal matrix with diagonal elements *d*_*ii *_= Σ*_j _w_ij _*and the elements *q_ij _*of *Q *defines the probability of a random hop from gene *i *to gene *j*. The matrix *Q *satisfies the probabilistic constraint Σ*_j _q_ij _*= 1. As a result, the transition matrix of the genes following the PPI interaction network is defined:

$$C_{g}\left[i,j\right]=Q-D^{-1}W$$

**Figure 2:**
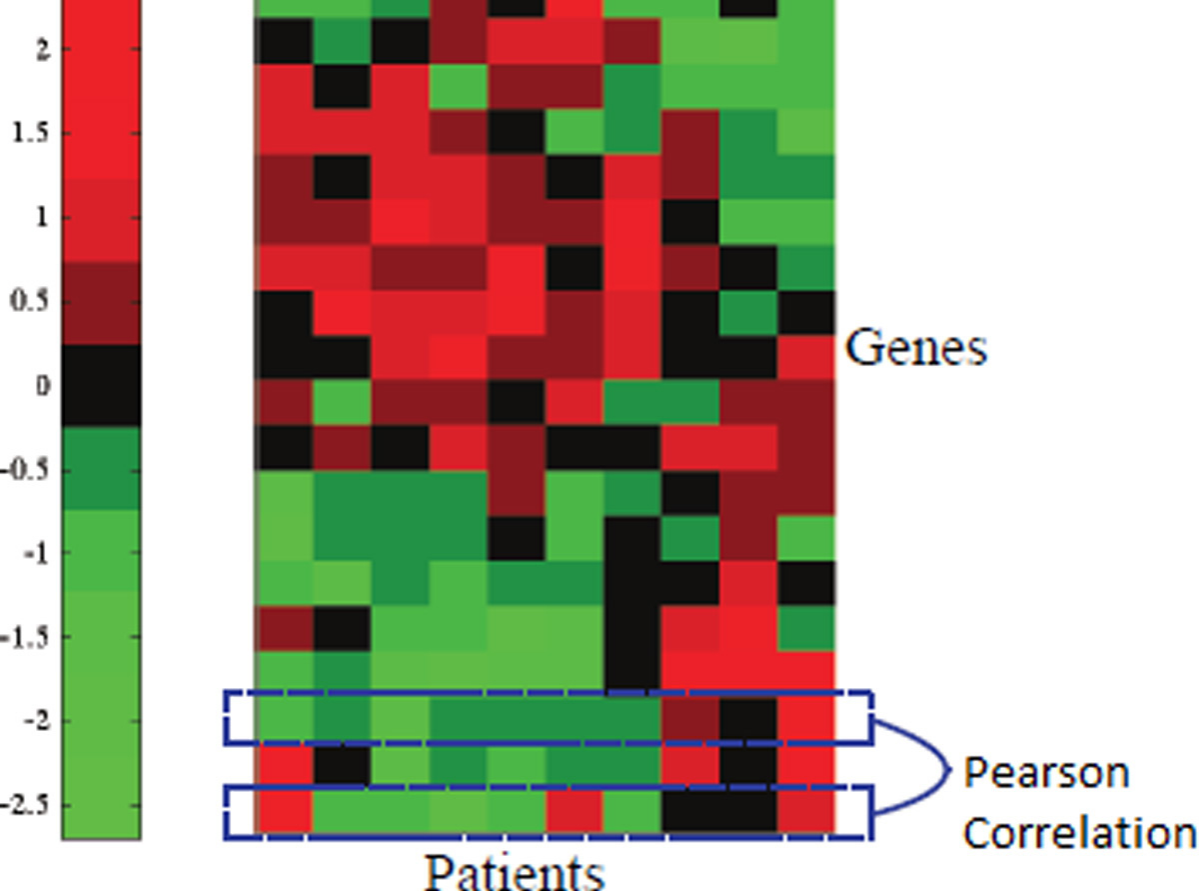
**Gene expression**.

Afterwards, gene expression data as shown in Figure 2 can come into play in the framework by constructing a gene correlation network. Each edge in the gene correlation network is encoded by matrix *H *whose elements *H*(*i, j*) represents the Pearson's correlation coefficient between the gene expression of gene *i *and gene *j*. To derive, we let *β_u _*to be the gene expression vector of gene *u *on the patients and so

$$H\left(u,v\right)=corr\left(\beta_{u},\beta_{v}\right)$$

$$=\frac{\text{Σ}t\in V\left(B_{u}\left(t\right)-\frac{1}{V}\right)\left(\beta_{v}\left(t\right)-\frac{1}{V}\right)}{\sqrt{\text{Σ}t\in V{\left(\beta_{u}\left(t\right)-\frac{1}{V}\right)}^{2}}\sqrt{\text{Σ}t\in V{\left(\beta_{v}\left(t\right)-\frac{1}{V}\right)}^{2}}}$$

where corr(X, Y) represents the Pearson correlation of random variable × and random variable Y. The intuition behind it is that the gene expression of two genes may be correlated to each other if they are both partcipating in the same cancer pathway. Next, normalization of matrix *H *to define the transition probability is performed as above which results in the following transition matrix of the genes for the gene correlation network:

$$C_{h}=T=D^{-1}H$$

#### Markov Chains

**Figure 3:**
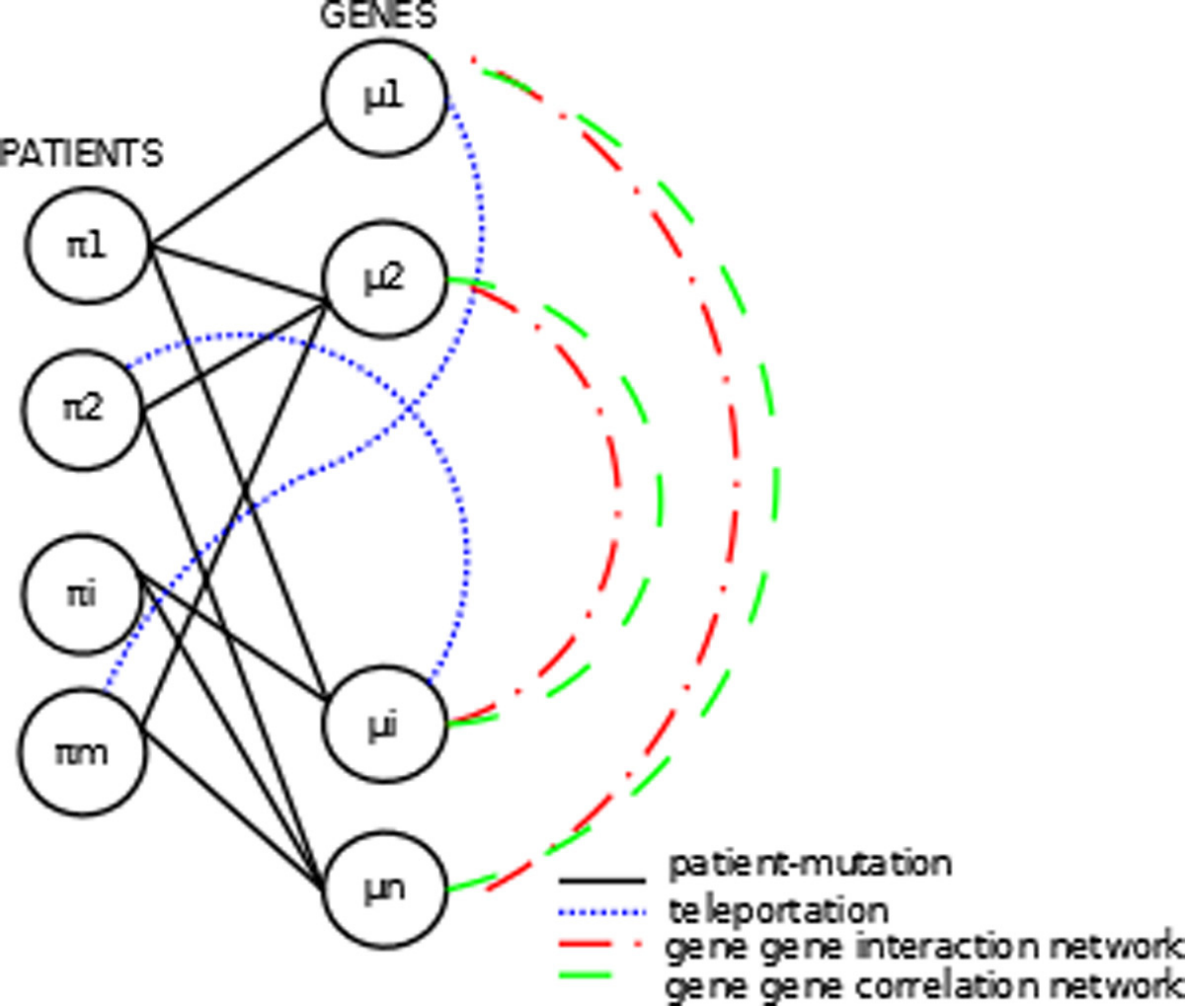
**Overall patient mutation network**.

In this section, a series of models using the transition probability matrices are defined. Figure 3 shows the overall models used in our framework.

1. Random Walk Multiplicative Model Gene Correlation Start(RW-MMGCS):

The model starts with a random walk on gene correlation network and followed by PPI network and then to patient and back to gene correlation network and iterate. The stationary distribution of the random walk is defined as follow:

(1) $$\begin{array}{c} \mu =C_{r}^{T}C_{c}C_{g}^{T}C_{h}^{T}\mu \\ \pi =C_{c}C_{g}^{T}C_{h}^{T}C_{r}^{T}\pi . \end{array}$$

2. Random Walk Multiplicative Model Gene Interaction Start(RW-MMGIS):

The model starts with random walk on gene gene interaction network and followed by gene correlation network and then forward to patient and back to PPI network and reiterate. The stationary distribution of the random walk is defined as follow:

(2) $$\begin{array}{c} \mu =C_{r}^{T}C_{c}C_{h}^{T}C_{g}^{T}\mu \\ \pi =C_{c}C_{h}^{T}C_{g}^{T}C_{r}^{T}\pi . \end{array}$$

3. Random Walk Additive Model (RW-AM):

This random walk framework is slightly different from the above random walk settings in the sense that the transition matrix for the mutations is defined as a linear combination of the transition matrix of gene gene interaction network, patient mutation profile and the gene correlation network with each transition matrix responsible for a part of the overall transition matrix. It is defined as follow:

(3) $$\begin{array}{c} \mu =\left(\alpha *C_{r}^{T}C_{c}+\beta *C_{g}^{T}+\gamma *C_{h}^{T}\right)\mu \\ 1=\alpha +\beta +\gamma \\ \pi =C_{c}C_{g}^{T}C_{h}^{T}C_{r}^{T}\pi . \end{array}$$

4. Random Walk Multiplicative Model Penalised:(RW-MMP)

In this model, the gene gene interaction network and gene correlation network is combined into one network. As described above, the gene gene interaction network is encoded by a weight matrix *W*, whose element *w_ij _*represents the interaction strength between gene *i *and gene *j *and the gene correlation network is weighted by matrix *H *whose elements *H_ij _*is the Pearson's correlation coefficients between the gene expression of gene *i *and gene *j*. Each edge in the PPI network encoded by matrix *W *is penalized by the expontential value of its corresponding gene correlation value weighted by matrix *H *divided by the average. Then the random walk starts with overall gene network and then to gene patient and then back to overall gene network and repeats. The following equations are justified.

(4) $$\begin{array}{cc} \sigma & =\text{mean of all entries of matrix H}\\ W_{ij} & =W_{ij}\text{exp}\left(H_{ij}/\sigma \right)\\ C_{g}\left[i,j\right] & =Q=D^{-1}W\\ \mu & =C_{r}^{T}C_{c}C_{g}^{T}\mu \\ \pi & =C_{c}C_{g}^{T}C_{r}^{T}\pi . \end{array}$$

5. Random Walk Multiplicative Model Average:(RW-MMA)

In this model, we take the average output from the random walk on gene gene interaction network, gene correlation network and patient mutation profile in each iteration. The algorithm is tabulated in Algorithm 1.

6. Random Walk Multiplicative Patient Profile Start(RW-MMPFS):

The model starts with random walk on gene-patient network, followed by PPI network and then to gene correlation network and then back to gene correlation network and iterate. The stationary distribution of the random walk is defined as follow:

(5) $$\begin{array}{c} \mu =C_{h}^{T}C_{g}^{T}C_{r}^{T}C_{c\mu }\\ \pi =C_{c}C_{g}^{T}C_{h}^{T}C_{r}^{T}\pi . \end{array}$$

**Algorithm 1 **Random Walk Multiplicative Model Average algorithm (RW-MMA)

  **procedure **Random Walk Multiplicative Model Average (*l, m, r, Cg, Cr, Cc, C_h_*)

    *R*_0 _*← *all entries are 1/n

    **for ***t *= 1 to max(*l,m,r*) **do**

      **if ***t <*= l then $R_{left}=C_{r}^{T}C_{c}*R_{t-1}$

      **end if**

      **if ***t <*= m then $R_{mid}=C_{g}^{T}*R_{t-1}$

      **end if**

      **if ***t <*= r then $R_{right}=C_{h}^{T}*R_{t-1}$

      **end if**

$$R_{t}=\frac{\left(\sigma_{t<=l}*R_{left}+\sigma_{t<=m}*R_{mid}+\sigma_{t<=r}*R_{right}\right)}{\sigma_{t<=l}+\sigma_{t<=m}+\sigma_{t<=r})}$$

      $\sigma_{t}<=x=1$ if t<=x and 0 otherwise

    **end for**

    **return **^*Rt*^

  **end procedure**

In the aftermath of defining various models, it remains to demonstrate that all the Markov chains in all the five proposed models are valid and all the corresponding transition matrices converge to unique stationary matrices which result in a unique eigenvector as our ranking vector in each model.

**Lemma: **All the above transition matrices define valid Markov Chains that converge to a unique stationary eigenvectors.

For the sake of simplicity, the proof of the model Random Walk Multiplicative Model Gene Interaction Start(RWMMGIS) is outlined as below, the rest of the models can be proved similarly. Convergence: To prove convergence, we must prove the Markov chain defined by the transition matrix $C_{r}^{T}C_{c}C_{h}^{T}C_{g}^{T}$ is irreducible and aperiodic. Notice that each mutation is permitted to teleport to any patient and each patient is permitted to teleport to any mutation with a small probability. Coupled with the definitions of *B_r _*and *B_c_*, all entries in matrix *C_r _*and *C_c _*are strictly positive. Since *C_h _*and *C_g _*are also positive stochastic with nonnegative entries, the transition matrix defined by $C_{r}^{T}C_{c}C_{h}^{T}C_{g}^{T}$ are all strictly greater than 0 in all entries. This proves that every state in the state space S can be reached from every other state in the state space in a finite number of moves with positive probability which proves irreducibility. For aperiodicty, notice the fact that each *P_ii _>*0 which implies that the minimum number of steps from each state i returning to itself is 1 which proves aperiodicity. Uniqueness: To prove uniqueness, notice that $C_{r}$ is a row stochastic matrix and hence $C_{r}^{T}$ is column stochastic, in addition, $C_{h}^{T}$, $C_{r}^{T}$, $C_{c}$, $C_{g}^{T}$ are all positive column stochastic and hence the product of positive column stochastic matrices is also positive column stochastic. By Perron-Frobenius Theorem, 1 is an eigenvalue of multiplicity one of the matrix $C_{r}^{T}C_{c}C_{h}^{T}C_{g}^{T}$ which is the largest and all the other eigenvalues are in modulus smaller than 1. Furthermore the eigenvector corresponding to eigenvalue 1 has all entries positive. In particular, for the eigenvalue 1 there exists a unique eigenvector with the sum of its entries equal to 1. This gives us a unique eigenvector as our rank for the genes. Similar arguments can be applied for the proof of the existence of our patient rank.

## Result

The data sets used for the experiment were taken from the study of Integrated Genomic Analyses of Ovarian Carcinoma led by the Cancer Genome Atlas. The associated results and discussions were published in Nature 2011 [29]. The analysis of 489 clinically annotated stage III-V HGS-OvCa samples and its corresponding normal DNA were reported in the article and posted on its associated website. The data incorporates the age at diagnosis, stage, tumour grade and surgical outcome of patients diagnosed with HGS-OvCa. We downloaded the TCGA-OV-mutations data and the unified expression profiles from the TCGA Data Portal website for our purpose. In the aftermath of data cleaning, we retain mutations containing insertion, deletion and alternation of base only. Finally a patient mutation profile table which comprises 316 patients and 8404 genes is obtained. Similar procedures were carried out on obtaining the gene expressions data from the website. Pearsons correlation coefficients are calculated on the gene expression data in pairwise fashion to obtain the gene correlation value between each pair of genes and the gene corelation graph is constructed. We utilized two different protein protein interaction networks for our experments. HumanNet is a probabilistic functional gene network which consists of 18,714 protein encoding genes and 476399 interactions between the genes of Homo sapiens. Pathway Commons is a collection of publicly available metabolic pathway database in conjunction with interactions from multiple organisms. It was filtered to retain human genes and interactions for the sake of our experiments. We obtained the required data through its web portal for download and query.

### Ground Truth Data

We compiled a set of genes published in various literature on several cancer studies which are certified to be ovarian cancer genes to be our ground truth cancer genes in the evaluation of our proposed models. Afterwards, the experiments on our five proposed models are run. A gene scoring vector (gene rank *µ*) for each of the six models is obtained. We then evaluate our proposed models by the rankings of the ground truth genes in each of the six proposed models' gene scoring vector *µ *and demonstrate the effects of integrating more background information in ranking. Precision/Recall graph and the top 25 genes appeared in each of the gene scoring vector (gene rank *µ*) of the five proposed models are presented in subsequent sections. Table 1 below tabulates the collection of ovarian cancer genes (ground truth genes) and the associated references.

**Table 1:** Ground Truth Genes.

GENE	Literatures
BRCA1	[12,13,29]

BRCA2	[14]

BMPR1A	[17,20]

BRIP1	[25]

MLH1	[15]

FHIT	[14,35]

TFRC	[16]

FGFR2	[18,19]

GATA3	[21]

MYST4	[34]

PTEN	[22]

FAS	[23,24]

RB1	[25]

SEPT9	[26]

YWHAE	[33]

TP53	[29]

PIK3CA	[27]

BRAF	[27]

KRAS	[27]

AIB1	[28]

MSH2	[15]

BMP4	[31,32]

TRIP1	[30]

MYC	[30]

EP300	[30]

### Experimental Results

We run the experiments on our six proposed models using the data set we obtained. In our experiments, we set *α *= 0.75. For the additive model (RW-AM), we set *α *= 0.3, *β *= 0.3 and*γ *= 0.4. Three benchmark models are utilized to evaluate our proposed models. The first one is frequency based in which each gene is awarded a rank in accordance with the occurence of mutation which means the higher the frequency of occurence of mutations on that gene, the higher rank will be awarded. The other two benchmark models are random walk based in which we perform random walk on gene correlation network (RW-GC) and patient mutation (RW-PM) network respectively and a gene scoring rank vector *µ *for each network is attained. We present the total number of appearances of ground truth genes in the top 1 percent of the gene rank *µ *of each model as follows in Table 2:

**Table 2:** Top 1 percent of the gene ranks.

Models	Numbers Of Appearance	Average Rank
RW-MMGIS	14	40

RW-MMGCS	17	38

RW-MMPFS	14	44

RW-AM	15	39

RW-MMP	16	40

RW-MMA	16	42

RW-GC	9	62

RW-PG	4	17

FREQUENCY BASE	4	18

The six proposed models outperform all the benchmark models. This can be demonstrated from the above table that the number of occurences of ground truth genes in the above six models outnumbers the three benchmark models. We found that incorporating heterogeneous sources of biological information enhances the performance of identifying ovarian cancer genes. In the nine models, RW-MMGCS yields the best performance, followed by RW-MMA and then RW-MMP and then RW-AM and then RW-MMGIS and then RW-MMPFS and then followed by three benchmark models at last: RW-GC, RW-PG and FREQUENCY BASE. Please note that a larger gap occurs between the results of two benchmark models with RW-GC outperforming RW-PG. This underscores that the gene expression data is more informative than patient mutation profile in locating ovarian cancer genes. All in all, integrating various heterogeneous sources of information helps in locating ovarian cancer genes.

### Evaluation

**Figure 4:**
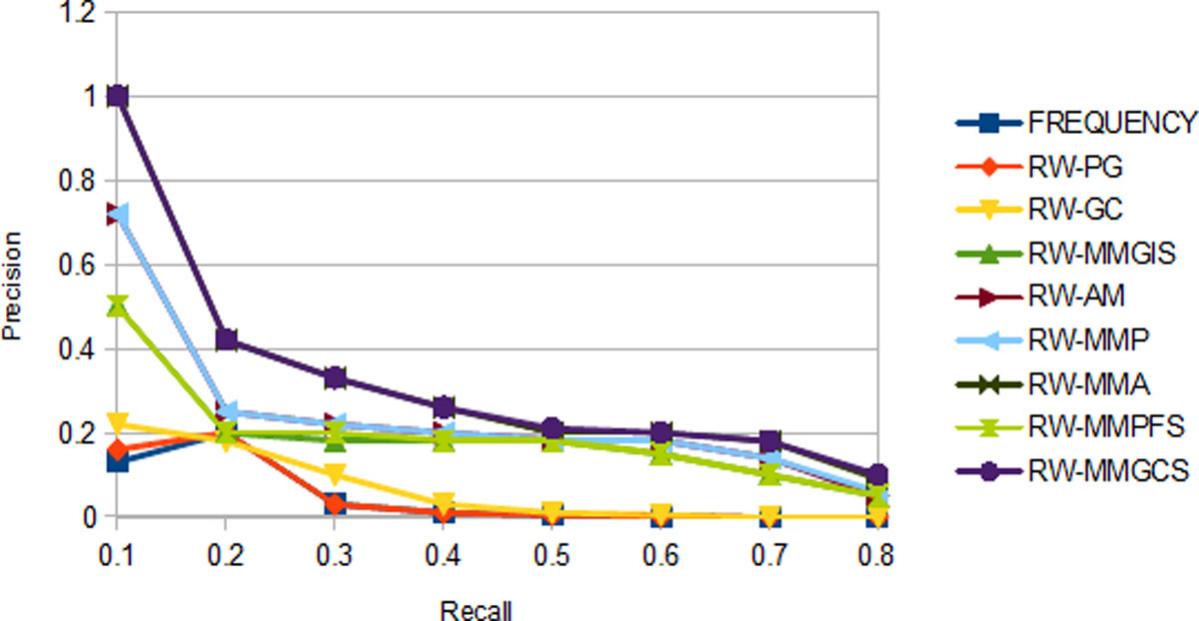
**Recall/precision**.

In this subsection, Precision/Recall graph by adjusting the threshold on the rank of the ground truth genes is presented. Precision is defined as the fraction of the ground truth genes among all genes ranked above each threshold. Recall is defined as the fraction of ground truth genes which are ranked above each threshold among all known ground truth genes. 25 ground truth genes in each experiment are used and the results are tabulated in Figure 4.

All six proposed models outperform the three benchmark models. RWMMGCS yields the best performance, followed by RW-MMA and then RW-MMP and then RW-MMGIS and then RW-AM. RW-MMGCS, RW-MMA and RW-MMP show a very high precision rate at recall rates running from 0.1 to 0.2. This demonstrates that they are able to locate several true positive genes (ground truth genes) in topmost positions within the ranked list. Since we use only 25 ground truth genes in our experiments, we expect to achieve a better result if more candidate cancer genes are included. Almost all the models decrease their performances monotonically towards the higher recall rate except FREQUENCY BASE and RW-PG in which their precision increases a little towards a little higher recall rate and then plummets sharply. This can be explained by the fact that these two models discover a multitude of false positive at low recall rate while they obtain a little better precision towards higher recall rate when they are able to rank a few ground truth genes below the top ranked genes. Above all, we demonstrate that the integration of more hetergeneous background information in the ranking helps achieve a better recall/precision rate.

**Figure 5:**
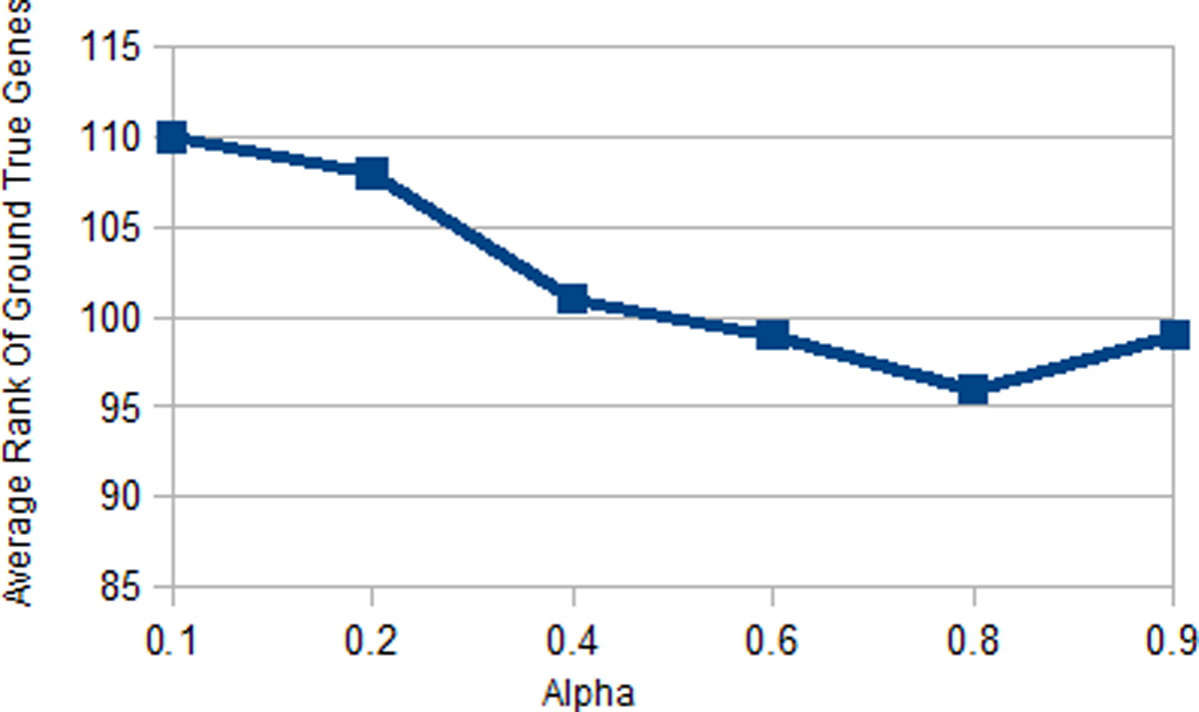
**Average rank of ground truth genes by adjusting teleportation parameter alpha**.

There is one parameter *α *in our proposed models (RWMMGCS, RW-MMA, RW-MMP, RW-MMGIS) which is the probability of teleportation of genes and patients. We performed an experiment on adjusting the value of *α *from 0 to 1 to inspect its relation to the average rank of the ground truth genes. The result is tabulated in Figure 5.

**Figure 6:**
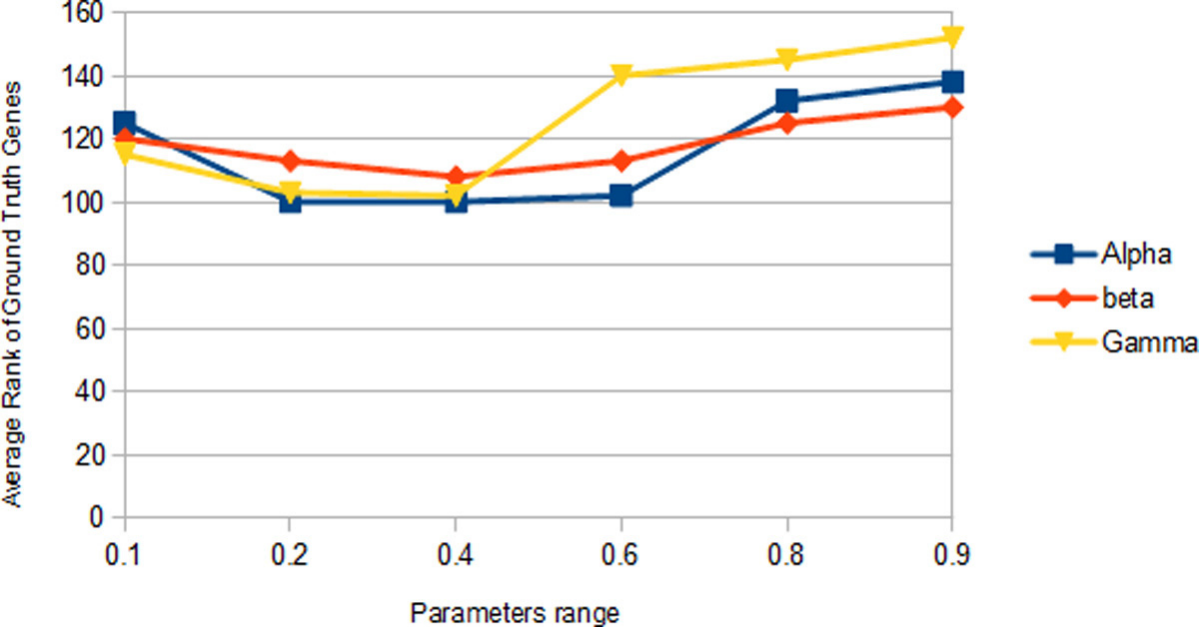
**Average rank of ground truth genes achieved by fixing each parameter in RW-AM**.

From above, the best *α *obtained is around 0.8 which achieves the lowest average ground truth genes ranking. Subsequently, in our additive model(RW-AM), we have three parameters *α, β, γ *that have to be determined. To evaluate these three parameters, we fix one of the parameters each time and adjust the other two parameters and record the best average ground truth genes ranking and the result is tabulated in Figure 6.

## Conclusion

In this paper, a Markov Chain Model for discovering important cancer genes through integration of heterogeneous sources of information are proposed: patient mutation profile, gene gene interaction network and gene correlation network in an unsupervised manner. Experimental results demonstrate that our proposed models outperform all benchmark models. Our future work will focus on developing graph Laplacian in learning cancer genes priority.

## Competing interests

The authors declare that they have no competing interests.

## Authors' contributions

Christopher Ma, Yixin Chen, Dawn Wilkins, Xiang Chen, and Jinghui Zhang planned the research. Christopher Ma prepared the computer code and ran the experiments. Christopher Ma, Yixin Chen, and Dawn Wilkins analyzed the results. Christopher Ma wrote the manuscript with assistance from Yixin Chen and Dawn Wilkins. All authors reviewed the manuscript before submission.

## Grants

The publication of this work was partly funded by NSF under award numbers EPS-0903787, and EPS-1006883, and MCB-1027989.

## Notes

This article has been published as part of *BMC Genomics *Volume 16 Supplement 9, 2015: Selected articles from the IEE International Conference on Bioinformatics and Biomedicine (BIBM 2014): Genomics. The full contents of the supplement are available online at http://www.biomedcentral.com/bmcgenomics/supplements/16/S9.
